# Development of the natural history component of an early economic model for primary sclerosing cholangitis

**DOI:** 10.1186/s13023-025-03658-8

**Published:** 2025-03-18

**Authors:** Christopher Bowlus, Cynthia Levy, Kris V. Kowdley, Nandita Kachru, Sushanth Jeyakumar, Yael Rodriguez-Guadarrama, Nathaniel Smith, Andrew Briggs, Mark Sculpher, Daniel Ollendorf

**Affiliations:** 1 University of California Davis School of Medicine, Sacramento, CA USA; 2 University of Miami Miller School of Medicine, Miami, FL USA; 3 Liver Institute Northwest, Seattle, WA USA; 4 Gilead Sciences, Inc, Foster City, CA USA; 5 Maple Health Group, LLC, New York, NY USA; 6 Occam Research, London, UK; 7 ICER Consulting Ltd, York, UK; 8 Center for the Evaluation of Value and Risk in Health, Tufts Medical Center, Boston, MA USA

**Keywords:** Primary sclerosing cholangitis, Economic, Natural history, Progression, Model, Survival

## Abstract

**Background:**

Primary sclerosing cholangitis (PSC) is a rare, chronic cholestatic disease that can progress to cirrhosis and liver failure. The natural history of PSC is variable as liver enzymes and liver symptoms fluctuate over time. Several drugs for PSC are under investigation, but there are currently no economic models to evaluate the cost-effectiveness and value of new treatments. The objective of this study was to develop an early economic model for PSC and validate the natural history component.

**Methods:**

A lifetime horizon Markov cohort model was developed to track the progression of adults with PSC with or without inflammatory bowel disease. Based on relevant literature and clinical expert advice, fibrosis staging was used to model disease progression. Evidence on disease progression, mortality, PSC-related complications, and secondary cancers was identified by literature searches and validated by interviews with clinical and cost-effectiveness modelling experts. Model outcomes were overall survival and transplant-free survival years, and the proportions of patients receiving liver transplants, 2nd liver transplants after recurrent PSC (rPSC), and developing rPSC after liver transplantation during their lifetime. Cumulative incidence of secondary cancers and quality-adjusted life-years (QALYs) were also tracked.

**Results:**

Model outcomes are in line with estimates reported in literature recommended by clinical experts. Overall survival (95% uncertainty interval [UI]) was estimated to be 25.0 (23.2–26.3) years and transplant-free survival was estimated to be 22.0 (20.2–23.6) years. The estimated proportion (95% UI) of patients receiving first liver transplants was 14.5% (11.6–17.1%), while the proportion of patients developing rPSC and receiving 2nd liver transplants after rPSC was 24.2% (20.4–28.0%) and 21.6% (12.9–29.7%), respectively. The cumulative incidence (95% UI) of cholangiocarcinoma, colorectal cancer, and gallbladder cancer were estimated at 5.2% (2.1–10.0%), 3.6% (1.4–5.4%), and 3.3% (1.2–7.6%), respectively. Discounted lifetime QALYs per patient (95% UI) were estimated at 16.4 (15.6–17.1).

**Conclusions:**

We have developed a model framework to simulate the progression of PSC with estimates of overall and transplant-free survival. This model, which calibrates well with existing estimates of disease progression, may be useful to evaluate the clinical and economic benefits of future treatments.

## Introduction

Primary sclerosing cholangitis (PSC) is a rare, chronic cholestatic disease of unknown cause characterized by liver inflammation, fibrosis, and destruction of intrahepatic and extrahepatic bile ducts [1]. Eventually PSC can result in cirrhosis and liver failure, with the 10-year survival rate ranging from 64% in the UK (with a median diagnosis age of 57 years) to 92–94% in Italy and Finland (median diagnosis age 40 and 41 years, respectively) [2–4]. Early-stage disease is usually asymptomatic, and symptoms typically manifest in the 3rd or 4th decade of life [1, 5]. Symptoms include abdominal pain, pruritus, fatigue, jaundice, fever, and weight loss [1, 6]. The disease affects primarily adults and, rarely, children, occurring twice as often in males than females [5, 6]. In the US, the age- and sex-adjusted incidence of PSC is 1.47 per 100,000 person-years [7]. 

There is a strong association between PSC and inflammatory bowel disease (IBD). In a US population cohort study, concomitant IBD was found in 75% of patients with PSC, with 86% of the IBD cases associated with ulcerative colitis [7]. In addition, approximately 13% of patients with PSC have autoimmune comorbidities other than IBD, including autoimmune thyroiditis, hepatitis, and pancreatitis, as well as rheumatoid arthritis and sarcoidosis [8]. Other serious secondary complications are associated with PSC such as liver failure, acute cholangitis (also known as ascending cholangitis), hepatocellular carcinoma, colorectal cancer (CRC), cholangiocarcinoma (CCA), and gallbladder cancer (GBC) [9, 10]. Patients with PSC have a 3- to 4-fold higher risk of any cancer compared with individuals without PSC, and the risk of CCA is over 200- to 500-fold higher [2, 11]. Patients with PSC and IBD have a 10-fold higher risk of developing CRC compared with the general population [9]. Risk of death is 3.5-fold higher in patients with PSC than patients without PSC [11], primarily due to cancer and liver failure [12]. Two population cohort studies have reported the time to PSC-related death or time to transplant or death [2, 13]. In these studies, the age at diagnosis was 40.6 years and 38.9 years, respectively, the time to PSC-related death was 23.1 years and 33.6 years, and the time to transplant or death was 19.8 years and 21.2 years [2, 13]. 

Since early disease is typically asymptomatic, the first sign of PSC is often abnormal liver enzymes, particularly elevation of alkaline phosphatase and gamma-glutamyl transferase [1, 10]. There are no autoantibodies indicative of PSC [1]. Definitive diagnosis of PSC is by exclusion in the absence of any other conditions that may cause secondary sclerosing cholangitis and is generally confirmed by multifocal strictures of intrahepatic or extrahepatic bile ducts visualized by magnetic resonance cholangiography [10]. 

The natural history of PSC is highly variable as liver enzymes and liver symptoms fluctuate over time and is complicated by the autoimmune and cancer comorbidities [7, 14]. This variability makes defining and assessing a disease progression pathway challenging. Although nearly all patients ultimately develop decompensated cirrhosis, disease progression is unpredictable as definitive predictive biomarkers are yet to be validated [7, 15]. Therefore, symptoms and complications are currently used as signs of progression [14]. 

There is currently no licensed medical therapy specifically for PSC. The only effective treatment is liver transplantation, which is needed by approximately 15% of patients [12]. Even after transplantation, approximately 20-30% of patients experience a recurrence of PSC within 10 years [16, 17]. Thus, effective medications for PSC are urgently needed, and several drugs with various mechanisms of action are under evaluation. Economic models will be needed to determine the long-term clinical and economic value of these treatments. However, there is currently a paucity of such economic models for PSC treatments. The objective of this study was to develop an early economic model framework for PSC and to validate the natural history component through discussions with clinical and health economic experts.

## Methodology

A probabilistic Markov cohort model was developed to track the progression of a mixed cohort of adults with PSC with or without IBD. A lifetime horizon was employed and a yearly discounting rate of 3% was utilized for QALY outcomes in line with standard practice in the United States [18]. A 1,000-iteration Monte Carlo simulation was used to capture parameter uncertainty using appropriate probability distributions for model inputs. Uncertainty metrics were sourced from the literature. All analyses were performed in Microsoft Excel.

### Validation of analytical approach

Given the limited published natural history models in PSC and owing to similar clinical characteristics between PSC and primary biliary cholangitis (PBC) [19], the model structure was based upon a similar model used in health technology assessment submissions for PBC [20]. Evidence to populate the model was established by literature searches and validated via interviews with 3 US clinical experts and 3 health economic experts. The interviews with clinicians were conducted to determine assumptions and evidence for disease progression and PSC-related complications. The interviews with the health economic experts were conducted to determine assumptions and inputs for the overall model structure.

## Model assumptions and inputs

**Fig. 1:**
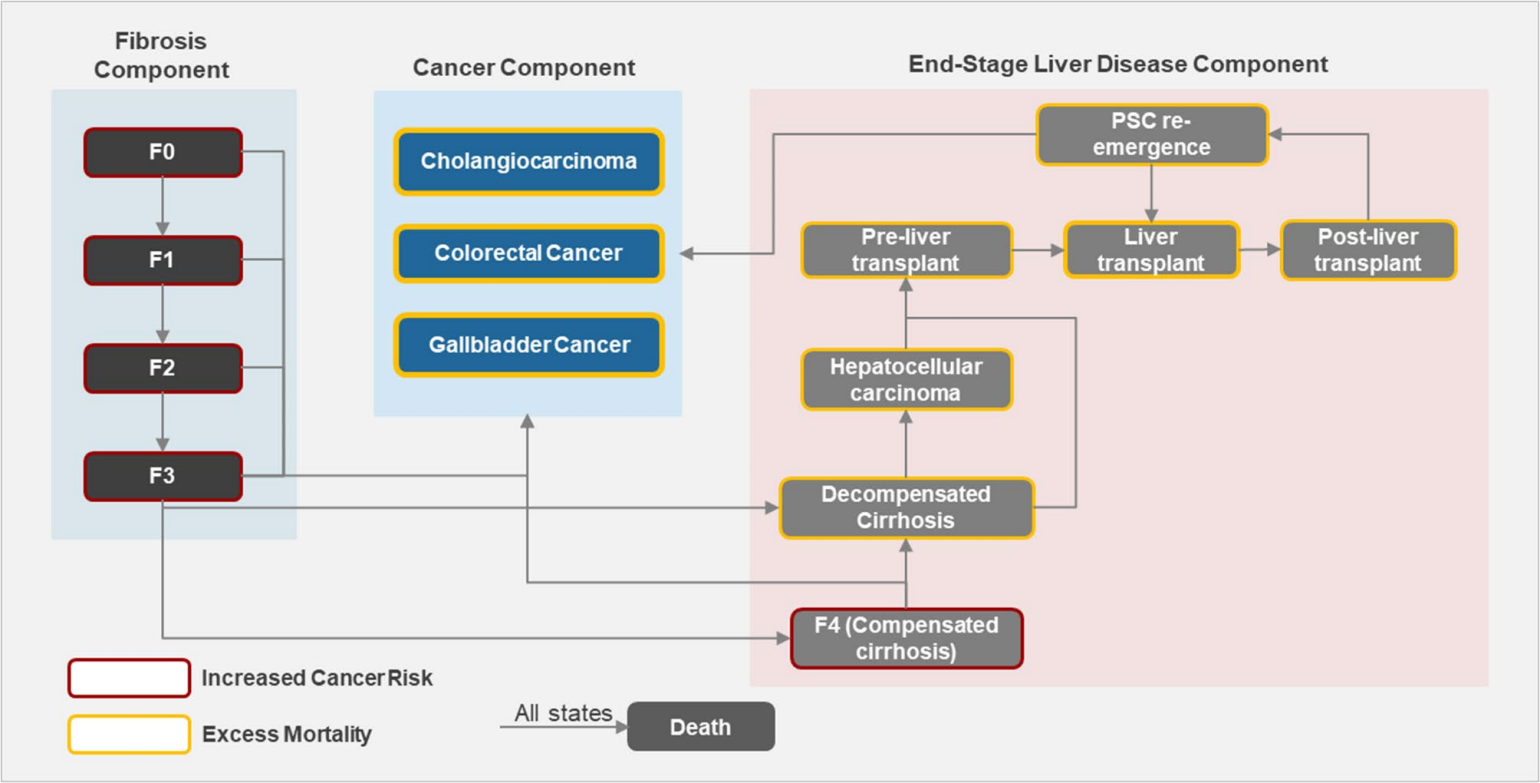
Modeled progression of disease. IBD, inflammatory bowel disease; PSC, primary sclerosing cholangitis

The natural history component of the model includes inputs for disease progression, mortality, and PSC-related complications, including secondary cancers. The population cohort was mixed in regard to presence or absence of IBD and fibrosis stage [21]. Patients enter the model at age 39 years, and 70% of the cohort had concomitant IBD as observed in a PSC population-based cohort study [13]. All patients were assumed to be initially pre-cirrhotic (F0-F3) and were distributed across individual fibrosis stages as reported previously (F0 = 9.83%, F1 = 19.65%, F2 = 27.75%, F3 = 42.77%) [22]. Initial distributions were implemented aiming at validation of the mix-cohort model results in comparison to published estimates. Additionally, reduction of liver fibrosis by one stage has been evaluated as a key efficacy endpoint in clinical trials of PSC [23–27]. As such, a progressive transition through fibrosis stages was assumed as depicted in Fig. 1. The inputs to derive transition probabilities for disease progression were determined from the literature and are shown in Table 1 [17, 20, 22, 28–33]. The model assumes no possibility of fibrosis regression. Based on the interviews with clinical experts, it is assumed that progression to decompensated cirrhosis can occur after F3 or F4 and that progression to hepatocellular carcinoma can occur only after developing compensated or decompensated cirrhosis (Fig. 1).

Complications included in the model were acute cholangitis and secondary cancers. The annual risk for acute cholangitis was assumed to be 1.76% based on published literature [13, 34]. Patients in all fibrosis stages were assumed to be at risk of acute cholangitis. As reported in the literature, PSC patients are at an increased risk of developing secondary cancers, particularly CCA, CRC, and GBC. The transition to these cancers were assumed to occur from all fibrosis stages and is not limited to advanced liver disease [9, 35, 36]. Secondary cancer model inputs are shown in Table 1.

No excess mortality was assumed with acute cholangitis based on clinical feedback. Annual mortality risks for advanced liver disease, liver transplantation, and secondary cancers were derived from the literature and are shown in Table 1.

Health-related quality of life weights (utilities) were sourced from the literature and were used to compute QALYs for each of the model health states. Inputs and sources for utilities are presented in Table 2.

### Model outcomes

The model tracks overall survival and transplant-free survival reported as years per patient, proportion of patients receiving liver transplants and 2nd liver transplants after recurrent PSC (rPSC), and proportion of patients developing rPSC after liver transplant. Cumulative incidence of secondary cancers is also reported. In addition to clinical outcomes, discounted QALYs per patient are reported. Clinical model outcomes were compared to key published estimates by adjusting model settings to study characteristics (age, proportion of people with IBD, and time horizon). For the comparison of rPSC and 2nd liver transplantation, the cohort was assumed to start the model in the respective health state. The selection of publications for comparison was based on considered sources of the model and clinical expert recommendations.

### Sensitivity analysis

One-way sensitivity analysis (OWSA) on overall survival and QALYs were conducted using the distributions and ranges for inputs described in Tables 1 and 2. Mean input estimates were varied using 95% uncertainty intervals (UI) or by +/-25% in the absence of variance data from the input source publications to test the impact of individual inputs upon selected outcomes.

**Table 1:** Disease progression inputs and literature sources

From	To	Model input: PSC only	Model input: PSC + IBD	PSC only: Lower bound	PSC only: Upper bound	PSC + IBD: Lower bound	PSC + IBD: Upper bound	Probability distribution	Literature Source
F0	F1	64.71%	64.71%	41.99%	87.42%	41.99%	87.42%	Beta	Bowlus et al. 2019 [22]
F1	F2	50.00%	50.00%	33.19%	66.81%	33.19%	66.81%	Beta	Bowlus et al. 2019 [22]
F2	F3	39.58%	39.58%	25.75%	53.42%	25.75%	53.42%	Beta	Bowlus et al. 2019 [22]
F3	F4 CC	31.08%	31.08%	20.54%	41.63%	20.54%	41.63%	Beta	Bowlus et al. 2019 [22]
F0-F4	CCA	248.58^†^	248.58^†^	153.57	293.73	153.57	293.73	Lognormal	Barner-Rasmussen et al. 2020 [2]
F0-F4	CRC	73.06^†^	73.06^†^	22.43	98.86	22.43	98.86	Lognormal	Barner-Rasmussen et al. 2020 [2]
F0-F4	GBC	36.53^†^	36.53^†^	-	54.79	-	54.79	Lognormal	Barner-Rasmussen et al. 2020 [2]
F3	DCC	1.03%	1.03%	0.77%*	1.28%*	0.77%*	1.28%*	Beta	Vilar-Gomez et al. 2018 [29]
F4 CC	HCC	2.00%	2.00%	1.50%*	2.50%*	1.50%*	2.50%*	Beta	Harnois et al. 1997 [33]
F4 CC	DCC	4.00%	4.00%	3.00%*	5.00%*	3.00%*	5.00%*	Beta	Tatar et al. 2020 [30]
HCC	LT	4.00%	4.00%	3.00%*	5.00%*	3.00%*	5.00%*	Beta	Wright et al. 2006 [28]
HCC	Death	43.00%	43.00%	32.25%*	53.75%*	32.25%*	53.75%*	Beta	Wright et al. 2006 [28]
DCC	LT	6.00%	6.00%	4.50%*	7.50%*	4.50%*	7.50%*	Beta	NICE 2017 [20]
DCC	Death	17.00%	17.00%	12.75%*	21.25%*	12.75%*	21.25%*	Beta	NICE 2017 [20]
LT	Death	9.21%	9.21%	4.61%	13.81%	4.61%	13.81%	Beta	Lindström et al. 2018 [32]
PLT	rPSC	14.34%	14.34%	11.45%	17.23%	11.45%	17.23%	Beta	Ravikumar et al. 2015 [37]
PLT	Death	10.23%	10.23%	7.40%	13.06%	7.40%	13.06%	Beta	Lindström et al. 2018 [32]
rPSC	LT	20.99%	20.99%	27.35%	47.95%	27.35%	47.95%	Beta	Ravikumar et al. 2015 [37]
rPSC	Death	10.98%	10.98%	4.33%	17.62%	4.33%	17.62%	Beta	Lindström et al. 2018 [32]
rPSC	CCA	248.58^†^	248.58^†^	153.57	293.73	153.57	293.73	Lognormal	Barner-Rasmussen et al. 2020 [2]
rPSC	CRC	73.06^†^	73.06^†^	22.43	98.86	22.43	98.86	Lognormal	Barner-Rasmussen et al. 2020 [2]
rPSC	GBC	36.53^†^	36.53^†^	-	54.79	-	54.79	Lognormal	Barner-Rasmussen et al. 2020 [2]

*Range derived in assumed variation of +/- 25% of mean value as no variance data was reported

^†^Cases per 1,000 PSC patients

CC, compensated cirrhosis; CCA, cholangiocarcinoma; CRC, colorectal cancer; DCC, decompensated cirrhosis; GBC, gallbladder cancer; HCC, hepatocellular carcinoma; IBD, inflammatory bowel disease; LT, liver transplant; PLT, post-liver transplant; PSC, primary sclerosing cholangitis; rPSC, reemergent PSC

**Table 2:** HRQoL weights per model health state and literature sources

Health State	HRQoL Weight	Range*		Literature Source
Fibrosis Stage - F0-F3	0.75	0.56	0.94	Assumption based on Kalaitzakis et al. 2016 [45]
Fibrosis Stage - F4	0.71	0.53	0.89	Kalaitzakis et al. 2016 [45]
DCC	0.66	0.49	0.82	Kalaitzakis et al. 2016 [45]
HCC	0.45	0.34	0.56	Wright et al. 2006 [28]
PLT	0.75	0.57	0.94	Kalaitzakis et al. 2016 [45]
rPSC	0.71	0.53	0.89	Assumed to be the same as F4
CCA	0.56	0.42	0.70	Zabernigg et al. 2012 [46]
CRC	0.68	0.51	0.85	Djalalov et al. 2014 [47]
GBC	0.56	0.42	0.70	Zabernigg et al. 2012 [46]
Acute cholangitis	0.24	0.56	0.94	Assumption based on Howard et al. 2006 [48]

*Range derived in assumed variation of +/- 25% of mean value as no variance data was reported

CCA, cholangiocarcinoma; CRC, colorectal cancer; DCC, decompensated cirrhosis; GBC, gallbladder cancer; HCC, hepatocellular carcinoma; HRQoL, health-related quality of life; PLT, post-liver transplant; PSC, primary sclerosing cholangitis; rPSC, reemergent PSC

## Results

### Model estimates

Deterministic and probabilistic results are presented in Table 3. Lifetime-estimates (95% UI) of overall survival and transplant free survival were estimated at 25.0 (23.2–26.3) and 22.0 (20.2–23.6) years per patient, respectively. The estimated proportion (95% UI) of patients in the model receiving first liver transplant was 14.5% (11.6–17.1%). The estimated proportions (95% UI) of patients developing rPSC after first liver transplant and receiving 2nd liver transplant after rPSC were 24.2% (20.4– 28.0%) and 21.6% (12.9–29.7%), respectively. The cumulative incidence (95% UI) of CCA, CRC, and GBC were estimated at 5.2% (2.1–10.0%), 3.6% (1.4–5.4%), and 3.3% (1.2–7.6%), respectively. Discounted QALYs per patient (95% UI) were estimated at 16.4 (15.6–17.1).

**Table 3:** Estimated outcomes for PSC generated from the economic model

Model outcomes	Deterministic mean	Probabilistic mean	Probabilistic 95% UI	
Overall survival, years	25.5	25.0	23.2	26.3
Transplant free-survival, years	22.7	22.0	20.2	23.6
Patients receiving first LT, %	15.0%	14.5%	11.6%	17.1%
Patients with rPSC relative to first LT, %	24.1%	24.2%	20.4%	28.0%
Patients receiving LT relative to rPSC, %	20.9%	21.6%	12.9%	29.7%
Cumulative incidence of CCA, %	5.0%	5.2%	2.1%	10.0%
Cumulative incidence of CRC, %	1.5%	3.6%	1.4%	5.4%
Cumulative incidence of GBC, %	0.8%	3.3%	1.2%	7.6%
QALYs	11.8	16.4	15.6	17.1

CCA, cholangiocarcinoma; CRC, colorectal cancer; GBC, gallbladder cancer; LT, liver transplant; PSC, primary sclerosing cholangitis; QALY, quality-adjusted life years; rPSC, recurring PSC; UI, uncertainty interval

### Comparison of model estimates with published literature

Model outcomes were in line with referenced sources for key outcomes as observed in Table 4. The model outcome for overall survival is similar to that reported in a Finnish population-based study of PSC (21.2 [95% UI 20.4–22.4] vs. 21.9 LY). Transplant-free survival was also similar to the Finnish study (19.4 [95% UI 18.4–20.9] vs. 18.3 LY) [2]. The estimated proportion of patients in the model receiving first liver transplant (11.3% [95% UI 9.4–13.8%]) is in line with that reported by Boonstra et al. [13] of 15.9%. An observational UK study of rPSC reported a 14.3% and 21% proportion of people developing rPSC after 1st liver transplantation and undergoing 2nd liver transplantation after rPSC, respectively [37]. Both estimates were similar to the model results for each parameter (13.2% [95% UI 11.1–15.2%] and 18.3% [95% UI 10.8–25.6%]). Cumulative incidence of secondary cancers was somewhat in line with what was reported by Barner-Rasmussen (2020). The authors found the cumulative incidence of CCA, CRC, and GBC to be 4.65%, 1.37%, and 0.68%, respectively [2]. Model results for CCA, CRC, and GBC were estimated at 6.7% (95% UI 4.1–9.0%), 4.5% (95% UI 1.7–7.0%), and 4.5% (95% UI 1.7–7.0%), respectively.

No studies reporting health-related quality of life in PSC were identified therefore no comparison to published data was possible.

**Table 4:** Comparison between key study estimates versus model generated outcomes

Study	Outcomes	Study estimate	Deterministic model outcome	Probabilistic model outcome (95% UI)
Barner-Rasmussen et al. [2]	Overall survival, years	21.9	21.6	21.2 (20.4–22.4)
	Transplant free-survival, years	18.3	20.2	19.4 (18.4–20.9)
	Cumulative incidence of CCA, %	4.65%	4.50%	6.7% (4.1–9.0%)
	Cumulative incidence of CRC, %	1.37%	1.33%	4.5% (1.7–7.0%)
	Cumulative incidence of GBC, %	0.68%	0.67%	4.5% (1.7–7.0%)
Boonstra et al. [13]	Patients receiving first LT, %	15.9%	12.1%	11.3% (9.4–13.8%)
Ravikumar et al. [37]	Patients with rPSC relative to first LT, %	14.3%	13.2%	13.2% (11.1–15.2%)
	Patients receiving LT relative to rPSC, %	21%	18.9%	18.3% (10.8–25.6%)

CCA, cholangiocarcinoma; CRC, colorectal cancer; GBC, gallbladder cancer; LT, liver transplant; rPSC, recurring PSC; UI, uncertainty interval

#### One-way sensitivity analysis

**Fig. 2:**
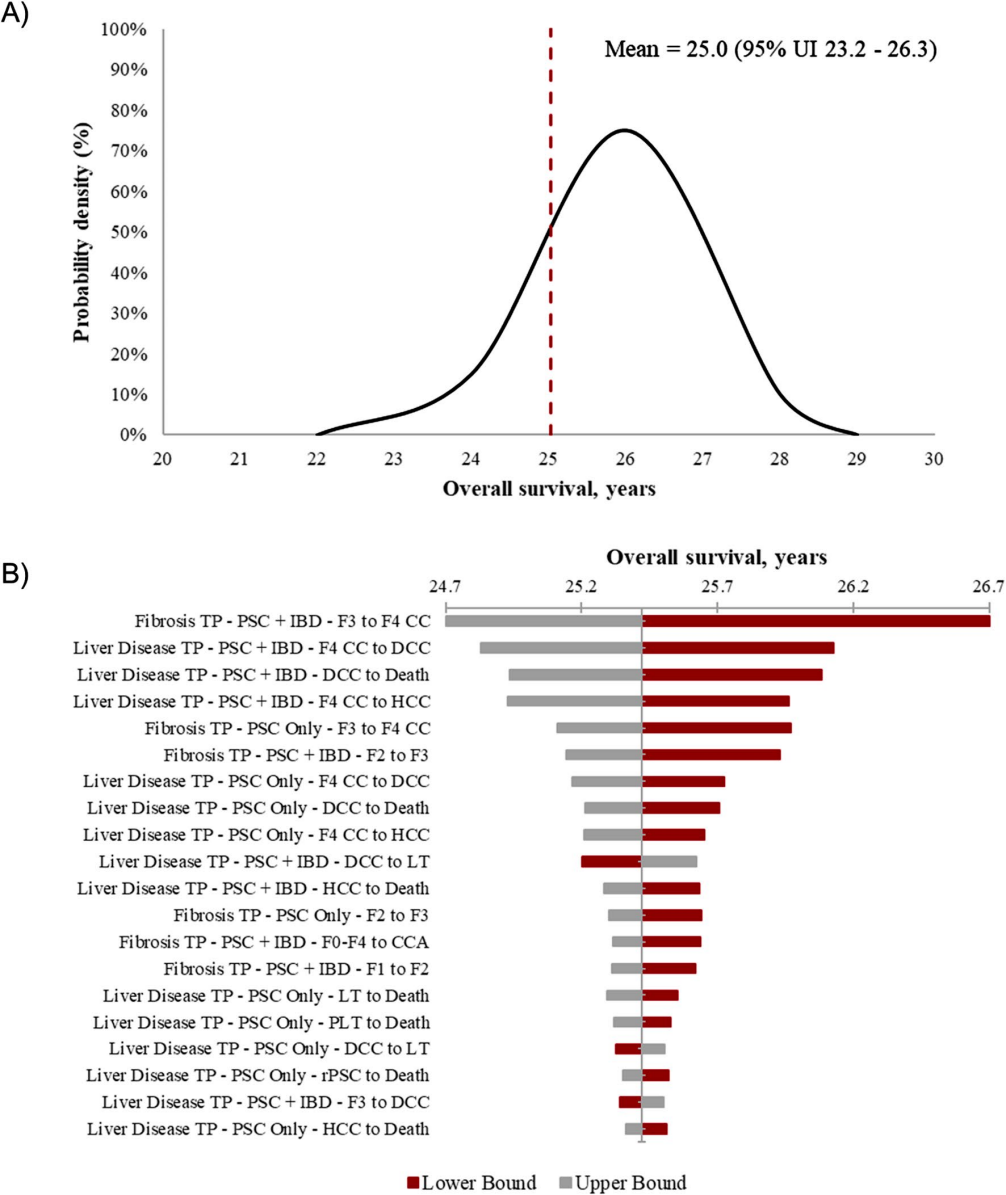
Sensitivity analysis of overall survival. (**A**) Probabilistic sensitivity analysis and (**B**) one-way sensitivity analysis. CC, compensated cirrhosis; CCA, cholangiocarcinoma; CRC, colorectal cancer; DCC, decompensated cirrhosis; GBC, gallbladder cancer; HCC, hepatocellular carcinoma; IBD, inflammatory bowel disease; LT, liver transplant; PLT, post-liver transplant; PSC, primary sclerosing cholangitis; rPSC, reemergent PSC; TP, transition probability; UI, uncertainty interval

**Fig. 3:**
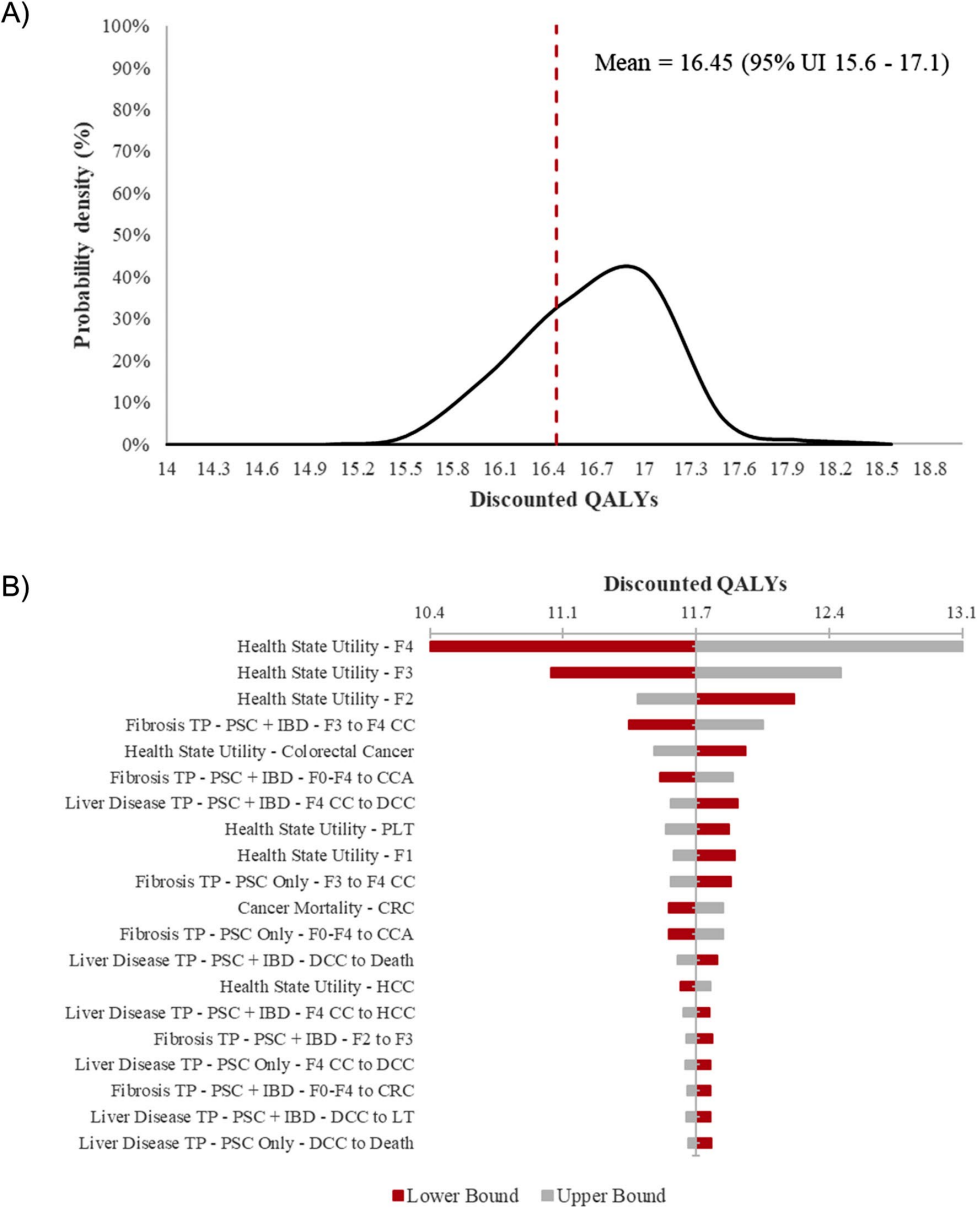
Sensitivity analysis of discounted quality-adjusted life years. (**A**) Probabilistic sensitivity analysis and (**B**) one-way sensitivity analysis. CC, compensated cirrhosis; CCA, cholangiocarcinoma; CRC, colorectal cancer; DCC, decompensated cirrhosis; GBC, gallbladder cancer; HCC, hepatocellular carcinoma; IBD, inflammatory bowel disease; LT, liver transplant; PLT, post-liver transplant; PSC, primary sclerosing cholangitis; rPSC, reemergent PSC; QALY, quality-adjusted life years; TP, transition probability; UI, uncertainty interval

The distribution of overall survival life years and discounted QALYs from the probabilistic sensitivity analysis are shown in Figs. 2 and 3 (Panel A), respectively. Tornado plots (Panel B) in Figs. 2 and 3 summarize the OWSA conducted on overall survival and discounted QALYs, respectively. The transition between fibrosis stage F3 and compensated cirrhosis (F4) in concomitant IBD patients appears to have the strongest influence upon overall survival (total change in value of 6%). The most influential input upon discounted QALYs is the utility tariff of F4 as shown in Fig. 3. The next most influential inputs on QALYs are the utility tariffs of F3 and F2, followed by the transition probability between F3 and compensated cirrhosis (F4).

## Discussion

Economic models provide helpful information for payers, decision makers, and healthcare providers on the comparative value of health care interventions. The natural history component of an economic model simulates the natural progression of an untreated disease using probabilities of transition from one health state to another, as well as clinical data on the impact of comorbidities and potential complications. Information on the natural history of PSC is limited, making development of an economic model challenging. Therefore, there is a paucity of such models for PSC. With guidance from literature sources, clinical experts, and cost-effectiveness modelling experts, the natural history component of an early economic model for PSC was developed using studies from many countries, making the model globally applicable. Future application of the model would be to assess the economic aspect of specific treatments, potentially through the use of hazard ratios for fibrosis progression, mortality, or other clinical outcomes. Fibrosis progression is a clinical endpoint in published and ongoing studies for PSC treatments [23–27], and until future indicators of progression are identified, fibrosis progression was deemed acceptable by clinical experts as the basis for the model structure of a cohort model in PSC.

A Markov model was chosen for the PSC economic model, similar to those described for economic modeling of PBC [20, 38]. Markov modelling is accepted and widely used in liver disease, although it may be limited in terms of being memoryless through transition between health states and model components. A patient-level simulation (PLS) analytic approach has also been used for PBC [39, 40] and may be appropriate for the associated complex assumptions needed to address the limitations of Markov modelling. Biomarker data could be leveraged in a PLS; however, the usefulness of PLS for PSC currently is somewhat limited by the lack of granularity linking the impact of PSC-related biomarkers and disease outcomes in PSC.

The foundational aspect of the model was the assumption of a simple linear progression of liver disease, despite the unpredictable trajectory of PSC. Since there are no defined milestones for disease progression, the interviewed experts agreed that transitioning between fibrosis states to end-stage liver disease was an acceptable assumption for modelling of PSC disease progression given the use of liver fibrosis progression as a key endpoint within recent PSC clinical trials. There are some caveats to this approach. First, fibrosis staging is determined by liver biopsy, which is not routinely used in the diagnosis of PSC. In addition, liver biopsy can be variable in sampling and does not capture the degree of fibrosis for the entire liver. Furthermore, there are multiple fibrosis staging systems besides the F0-F4 system used in the current model, such as the 7-stage Ishak fibrosis score [41]. Thus, to use the current model for evaluating the value of a particular treatment, biopsy data would need to be available and fibrosis staging would need to be captured using the F0-F4 staging system. Measurement of liver stiffness through imaging techniques such as magnetic resonance elastography or ultrasound elastography has emerged as a non-invasive method to assess the degree of fibrosis [42]. In addition, a number of blood-based tests that detect biomarkers of fibrosis or liver function are available, but these tests cannot yet reliably differentiate between different stages of fibrosis and need further validation for use in PSC [43]. As more data for the change in liver stiffness over time and its association with outcomes of interest become available, and blood-based tests become more accurate, the model could potentially be refined using non-invasive techniques instead of histology to approximate fibrosis staging. Further research and alternative measures of fibrosis (e.g., fibroscan) in earlier stages of the disease (F0-F3) may also support refining the definition of health states in these earlier stages.

Although the frequency of liver transplant generated by the model fell within the range reported in the literature, the variability in liver transplant frequency may be related, among other things, to complications related to acute cholangitis. This could be true because (1) occasionally an episode of cholangitis can lead to decompensation in a cirrhotic patient and (2) currently, the United Network for Organ Sharing accepts applications for Model for End Stage Liver Disease (MELD) exception points based on recurrent cholangitis. Physicians with patients who have had two or more episodes could apply for additional MELD points, bringing them higher up on the transplant list, thus leading to an earlier liver transplantation with MELD higher than their actual biological MELD. In addition, the distinction between orthotropic or living donor liver transplantation is not currently differentiated in the model. Future application of the model may want to account for any differential survival and recurrence based on the different types of transplants [44]. 

### Limitations

One of the challenges in developing the model was the lack of published data to inform specific aspects of the natural history model. For example, the lack of fibrosis staging and transition among patients with concomitant IBD required an assumption of being equivalent to patients without IBD. Additionally, some inputs for modelling advanced liver disease were drawn from other chronic liver diseases (i.e., PBC, hepatitis C). We acknowledge such assumptions as a model limitation. Nonetheless, sensitivity analyses show the model is robust as outcomes were similar to published estimates. Subtle discrepancies between model outcomes and published estimates may be due to differences in population, or different clinical management for patients requiring liver transplantation. Overall, the model outcomes seemed to be in line with key published literature.

Additional components of economic modelling include treatment benefits and harms, treatment and disease-related costs, treatment and disease-related health care resource use, and impacts of treatment and disease on quality of life (utility values). More research specific to PSC is also needed in these areas to facilitate development of the full economic model.

## Conclusions

We have developed an early economic model to simulate the progression of PSC with estimates of overall and transplant-free survival. Incorporation of costs and quality of life data for new treatments into this natural history model will allow for cost-effectiveness analyses similar to other disease models developed for liver diseases and PBC.

## Data Availability

All data supporting the findings of this study are available within the paper.

## Abbreviations

CC: Compensated cirrhosis
CCA: Cholangiocarcinoma
CRC: Colorectal cancer
DCC: Decompensated cirrhosis
GBC: Gallbladder cancer
HCC: Hepatocellular carcinoma
IBD: Inflammatory bowel disease
LT: liver transplant
PLT: Post-liver transplant
PSC: primary sclerosing cholangitis
rPSC: Reemergent PSC
UI: Uncertainty interval
